# DREAM regulates BDNF-dependent spinal sensitization

**DOI:** 10.1186/1744-8069-6-95

**Published:** 2010-12-18

**Authors:** Ivan Rivera-Arconada, Tomaso Benedet, Carolina Roza, Begoña Torres, Jorge Barrio, Agnieszka Krzyzanowska, Carlos Avendaño, Britt Mellström, José A Lopez-Garcia, José R Naranjo

**Affiliations:** 1 Department of Physiology, Faculty of Medicine, University of Alcala, 28871 Madrid, Spain; 2 Department of Molecular and Cellular Biology, National Centre for Biotechnology, C.S.I.C., Darwin 3, 28049 Madrid, Spain; 3 CNB-CIBERNED, 28049 Madrid, Spain; 4 Department of Anatomy, Histology and Neurosciences, Faculty of Medicine, Arzobispo Morcillo s/n, University Autónoma de Madrid, 28029 Madrid, Spain

## Abstract

**Background:**

The transcriptional repressor DREAM (downstream regulatory element antagonist modulator) controls the expression of prodynorphin and has been involved in the modulation of endogenous responses to pain. To investigate the role of DREAM in central mechanisms of pain sensitization, we used a line of transgenic mice (L1) overexpressing a Ca^2+^- and cAMP-insensitive DREAM mutant in spinal cord and dorsal root ganglia.

**Results:**

L1 DREAM transgenic mice showed reduced expression in the spinal cord of several genes related to pain, including prodynorphin and BDNF (brain-derived neurotrophic factor) and a state of basal hyperalgesia without change in A-type currents. Peripheral inflammation produced enhancement of spinal reflexes and increased expression of BDNF in wild type but not in DREAM transgenic mice. The enhancement of the spinal reflexes was reproduced *in vitro *by persistent electrical stimulation of C-fibers in wild type but not in transgenic mice. Exposure to exogenous BDNF produced a long-term enhancement of dorsal root-ventral root responses in transgenic mice.

**Conclusions:**

Our results indicate that endogenous BDNF is involved in spinal sensitization following inflammation and that blockade of BDNF induction in DREAM transgenic mice underlies the failure to develop spinal sensitization.

## Background

Transcriptional repressor activity of DREAM depends on their high affinity Ca^2+^- dependent binding as a heterotetramer to DRE (downstream regulatory element) sites in target genes [1-4]. Increased levels of intracellular Ca^2+ ^result in DREAM unbinding from DNA and transcriptional derepression [1]. Binding to DRE sites is controlled also by the interaction with other nucleoproteins [5,6]. DREAM mutants unable to respond to Ca^2+^, cAMP and/or to establish protein-protein interactions, function as cross-dominant constitutively active mutants (daDREAM) and repress permanently target genes in vivo [7,8]. Several genes have been shown to be regulated by DREAM, including prodynorphin, c-fos [1], AA-NAT, ICER [3], and BDNF [9] NCX-3 [8] and several cytokines in T lymphocytes [7]. DREAM, also known as calsenilin or KChIP-3 (K^+ ^channel interacting protein 3), interacts with presenilins or Kv4 potassium channels, respectively [10,11].

Genetic ablation of DREAM in DREAM^-/- ^mice results in increased thresholds for noxious stimuli that have been associated to increased prodynorphin gene expression and to reduction in A-type currents (I_A_) in spinal cord neurons [12-14]. However, reduction of A-type currents in spinal cord neurons of Kv4.2 deficient mice are associated with thermal and mechanical hyperalgesia and reduced responses to inflammation [15].

BDNF is implicated in the maintenance of peripheral sensory neurons during development and in the regulation of synaptic plasticity and long-term potentiation in the adult brain and spinal cord [16-19]. Expression of the BDNF gene depends on several regulatory regions [20]. Activity-dependent BDNF induction, following pain stimulation, is mainly controlled by regulatory elements in exon III in the rat gene. This includes, a hemi-palindromic CRE site that mediates CaMK IV-dependent transactivation by CREB/CBP following neuronal depolarization [21,22], two Ca^2+^-responsive elements, the CaRE sites, that bind the calcium responsive factor (CaRF) [23] and a DRE site that binds the transcriptional repressor DREAM [9].

Here we used transgenic mice expressing a cross-dominant constitutively active DREAM mutant to further analyze the functional role of DREAM in pain transmission and sensitization. Behavioral studies revealed that DREAM transgenic mice possess high sensitivity to thermal and chemical noxious stimuli and reduced hyperalgesic response to inflammation. Electrophysiological studies performed in isolated spinal cord of DREAM transgenic mice indicate the absence of hyperreflexia, a sign of sensitization [24], in response to persistent activation of nociceptive afferents. Quantitative real time-PCR showed that basal and inducible expression of BDNF is reduced in spinal cord and dorsal root ganglia (DRG) from DREAM transgenic mice. Though expression of the constitutively active DREAM mutant might affect the expression of several downstream genes, BDNF supplementation is enough to restore the capability of the spinal cord of DREAM transgenic mice to develop hyperreflexia.

## Results

### Characterization of L1 daDREAM transegenic mice

**Figure 1:**
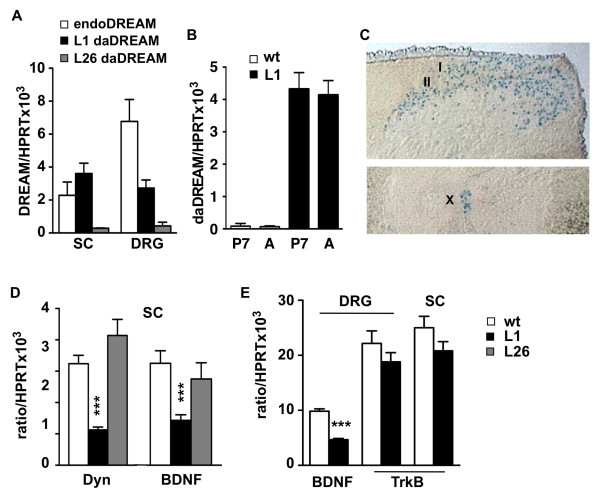
**Expression of DREAM, daDREAM and DREAM targets in spinal cord and dorsal root ganglia**. (A) Quantitative real-time PCR analysis of the expression of dominant active DREAM (daDREAM) in spinal cord (SC) and dorsal root ganglia (DRG) in transgenic L1 and L26 mice. For comparison, expression levels of endogenous DREAM (endoDREAM) are shown. (B) Comparison of the expression of dominant active DREAM mutant in spinal cord at P7 and adult in wild type (wt) and L1 mice. (C) β-galactosidase positive cells show the distribution of the transgene in lamina I-II and X in the spinal cord of L1 mice. (D) Quantitative real-time PCR analysis of the expression of prodynorphin (Dyn) and brain-derived neurotrophic factor (BDNF) in spinal cord from wild type (wt) and transgenic L1 and L26 mice. (E) Quantitative real-time PCR analysis of the expression of BDNF mRNA and its receptor, tyrosine receptor kinase B (TrkB) in DRG and spinal cord from wild type (wt) and L1 mice. The mRNA levels in A, B, D and E are expressed as ratio to HPRT mRNA levels. Data are mean ± SEM (n = 6-9 in A and B and 5-7 in D and E). *** *P *< 0.001.

Regulation of prodynorphin gene expression by DREAM has been associated with changes in the response to noxious stimuli [12,13] and learning [14]. To specifically analyze the role of DREAM in the molecular pathways that control the response to pain we used a line of transgenic mice (L1) expressing a cross-dominant constitutively active DREAM mutant (daDREAM) in neurons under the control of the CamKIIα promoter [25]. The ratio of daDREAM mRNA to endogenous DREAM was 1.6 to 1 and 1 to 3 in spinal cord and DRG, respectively (Figure 1A), indicating that in both areas the expression of the dominant mutant is enough to block endogenous DREAM-dependent derepression [7,8]. Expression of daDREAM in the spinal cord of L1 mice was observed early after birth and at postnatal day 7, daDREAM levels were not different from those in adult mice (Figure 1B). Another DREAM transgenic line (L26), with similar high expression of daDREAM in telencephalic areas as L1 (data not shown) but with very low expression in spinal cord and DRG (Figure 1A), was included in some experiments as a negative control. In transgenic L1 mice, expression of β-galactosidase, used as reporter gene in the bicistronic transgenesis cassette, could be observed in many neurons across all laminae of the spinal cord, with greater density in the dorsal horn and laminae X (Figure 1C). Expression of daDREAM protein in L1 mice resulted in a significant reduction in the basal levels of prodynorphin and BDNF mRNA in the lumbar spinal cord (Figure 1D). Expression of BDNF was reduced also in DRG, while levels of the TrkB receptor were not modified in the spinal cord or DRG (Figure 1E). Accordingly, no significant changes in dynorphin or BDNF expression were observed in spinal cord from line 26 mice (Figure 1D).

### Basal hyperalgesia and delayed sensitization to noxious stimuli in daDREAM transgenic mice

**Figure 2:**
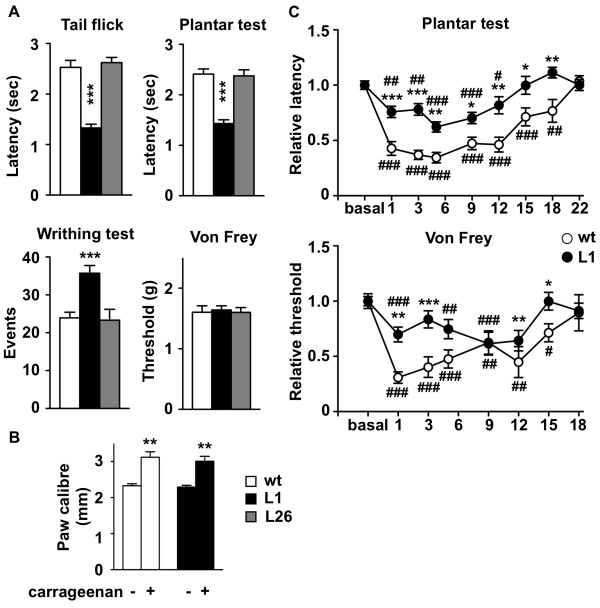
**Basal hyperalgesia and impaired sensitization after inflammation in transgenic mice**. (A) Basal sensitivity to nociceptive stimulation in the indicated tests in wild type (wt) and transgenic L1 and L26 mice. (B) Inflammatory response in wild type (wt) and transgenic L1 mice 24 h after intraplantar injection of carrageenan. (C) Nociceptive thresholds in the plantar and the Von Frey tests before and at the indicated days after intraplantar injection of complete Freund's adjuvant (CFA) in wild type (wt) and transgenic L1 mice. * Refers to comparison with wild type and # refers to comparison with day 0. Statistically significant differences between groups (n = 10) were measured by One-way ANOVA with Bonferroni post test. * or #, *P *< 0.05; ** or ##, *P *< 0.01; ***, *P *< 0.001.

Both transgenic lines developed normally to adulthood but line 1 mice showed enhanced response to thermal and visceral noxious stimuli in basal conditions compared to wild type littermates or L26 mice, however, response to mechanical stimulation was not different in L1 and wild type mice (Figure 2A). Intraplantar injection of complete Freund's adjuvant (CFA) or carrageenan produced similar redness and paw inflammation in wild type and L1 mice (Figure 2B). At 24 hours after CFA, wild type mice developed a pronounced thermal hyperalgesia (withdrawal latency of 1.29 ± 0.02 seconds), which lasted 18 days (Figure 2C). On the contrary, thermal thresholds were slightly modified in transgenic mice 24 hours after CFA and the hyperalgesic response was observed only up to day 12 after CFA (Figure 2C). Similar results were obtained when we tested for mechanical sensitivity. Wild type mice displayed a strong hyperalgesic response starting from day 1 after CFA injection, while in transgenic mice the hyperalgesia was less pronounced (Figure 2C). These data suggest that daDREAM mice display impaired response to inflammatory pain with milder and shorter-lasting hyperalgesia compared to wild type mice.

### Increased spinal reflexes in daDREAM mice

**Figure 3:**
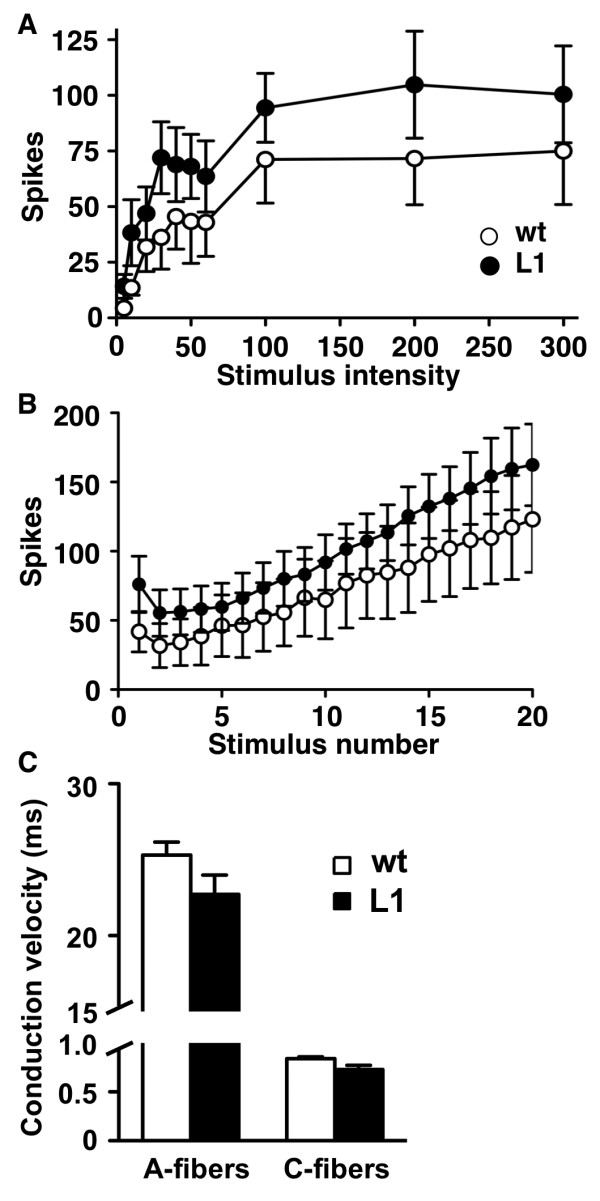
**The basal responsiveness of spinal cords from L1 mice is increased**. Comparison of ventral root responses to dorsal root stimulation obtained from wild type (wt) (n = 10) and L1 (n = 11) mice. (A) The intensity-response curve shows the number of spikes elicited by single stimuli of increasing intensities. Responses in L1 mice were larger (overall ANOVA, *P *< 0.01). (B) The wind-up curve shows number of spikes in ventral root recordings to trains of stimuli at C-fibre intensity (1 Hz). Responses in L1 mice were greater (overall ANOVA, *P *< 0.001). (C) Comparison of the conduction velocity in A- and C-fibers in wild type (wt) and L1 mice.

Segmental spinal cord circuits underlay motor withdrawal reflexes to nociceptive stimuli such as those used in behavioural pain testing. Therefore we analyzed dorsal root-ventral root reflexes (DR-VRRs) in wild type and transgenic mice using a spinal cord *in vitro *preparation from P9 mice [26]. Consistent with a basal hypersensitivity, responses to single stimuli of a wide range of intensities activating A- and C-fibres were increased in L1 mice (Figure 3A). The responses to trains of stimuli of C-fibre intensity consistently led to larger spike counts in L1 mice, while the slope of the resulting wind-up effect was not modified (Figure 3B). In contrast to the increased responsiveness of the spinal cord in L1 mice, nerve conduction velocities and electrical thresholds for A- and C-fibres were not significantly different from those of wild type mice (Figure 3C). These results suggest that the basal hyperalgesia observed in L1 mice may be related to an enhanced spinal processing of afferent signals rather than changes in afferent fibres.

### A-type currents in spinal cord neurons from L1 mice are normal

A-type currents have been associated with neuronal plasticity in the hippocampus [27] and the spinal cord [15]. The Ca^2+^-insensitive DREAM/KChIP3 mutant has been previously shown to affect gating of Kv4 potassium channels *in vitro *[11] and a weak change in A-type currents has been reported in DREAM deficient neurons [12]. We investigated whether a change in A-type currents in dorsal horn neurons from L1 mice could contribute to the basal hyperalgesia observed in daDREAM mice.

**Figure 4:**
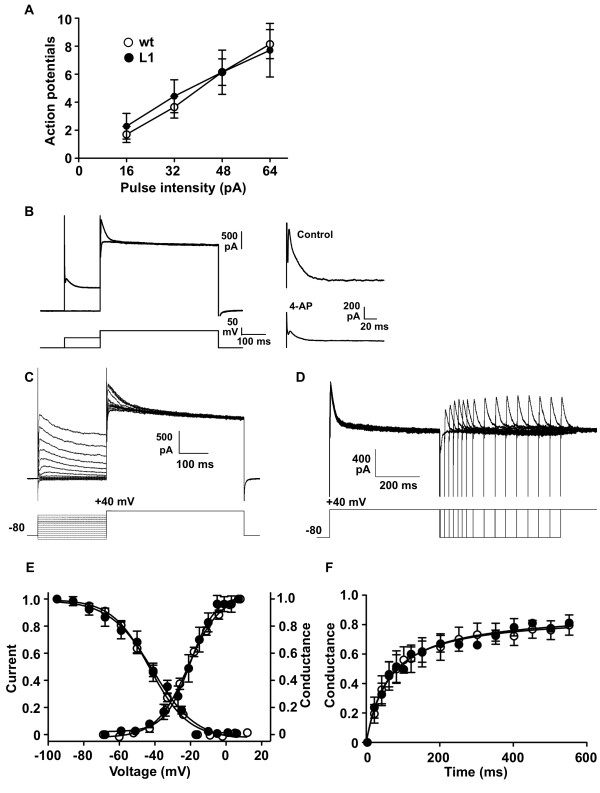
**General excitability and I_A _currents in spinal cord are identical in wild type and L1 mice**. (A) Mean number of action potentials obtained in current clamp recordings in response to depolarizing current pulses of different intensities. Wild type (wt) and L1 mice show similar behaviour. (**B**) The two-step protocol used to isolate I_A _current. Superimposed current (upper) and voltage (lower) traces (left) and current isolated by subtraction in control (upper) and after perfusion of 4-AP (lower) recordings (right) is shown. (**C **and **D**) Recordings obtained from dorsal horn neurons in control conditions show voltage-dependency of inactivation (**C**) and recovery from inactivation (**D**) of transient currents. (E, F) Analysis of the I_A _current in dorsal horn neurons in wild type (wt) and L1 mice. Transient currents show the same characteristics in both groups in terms of activation (E, right axis conductance) and inactivation kinetics (E, left axis current) and recovery from inactivation (conductance) (F). Graphs in E show pooled data of dorsal horn neurons from wild type (n = 18) and L1 (n = 6) mice. Graphs in F were obtained from neurons from wild type (n = 5) and L1 (n = 3) mice.

Neurons from wild type and L1 mice were recorded in current clamp mode to compare their general state of excitability. No differences were found in resting potential, input resistance and action potential threshold and amplitude (Table 1). Intracellular depolarizing current pulses of 500 ms and increasing intensities produced similar numbers of action potentials in both groups (Figure 4A), suggesting that the intrinsic excitability of transgenic neurons was essentially unchanged. Voltage clamp recordings in dorsal horn neurons allowed the isolation of A-type currents, which were similar in wild type and L1 mice (Figure 4B-D). No differences between groups were found in the voltage dependent activation and inactivation curves, current density and reactivation (Figure 4E, F and Table 1). These data indicate that daDREAM does not function as a dominant mutant for the spinal modulation of Kv4 potassium channel activity *in vivo*, and it is therefore unlikely that the basal hyperalgesia observed in L1 mice is related to changes in A-type currents.

**Table 1:** Basic electrophysiological properties (current-clamp) and major traits for transient (I_A_) currents (voltage-clamp) of neurons from wild type and transgenic mice

	Wild type	Transgenic
**Current-clamp**		
Number of neurons	19	8
RMP^a ^(mV)	-55.7 ± 0.8^f^	-53.9 ± 1.2
Rin^b ^(MΩ)	355 ± 20	387 ± 57
AP-A^c ^(mV)	65 ± 2	68 ± 3
AP-T^d ^(mV)	-39.7 ± 0.7	-40.1 ± 1.3
**Voltage-clamp**		
Number of neurons	18	7
Max current density (pA/pF)	15.3 ± 3.3	14.5 ± 8.4
Time constant (ms)	15.9 ± 1.5	15.7 ± 2.8
Activation; V_50_^e ^(mV)	-22.5 ± 2.1	-20.1 ± 2.6
Activation; slope factor	8.4 ± 0.7	6.5 ± 0.7
Inactivation; V_50_^e ^(mV)	-43.9 ± 1.7	-40.1 ± 2.5
Inactivation; slope factor	-10.5 ± 1.0	-9.5 ± 0.9

^a ^resting membrane potential; ^b ^input resistance; ^c ^action potential amplitude; ^d ^action potential threshold; ^e ^voltage of half maximal activation or inactivation; ^f ^mean ± SEM.

**Figure 5:**
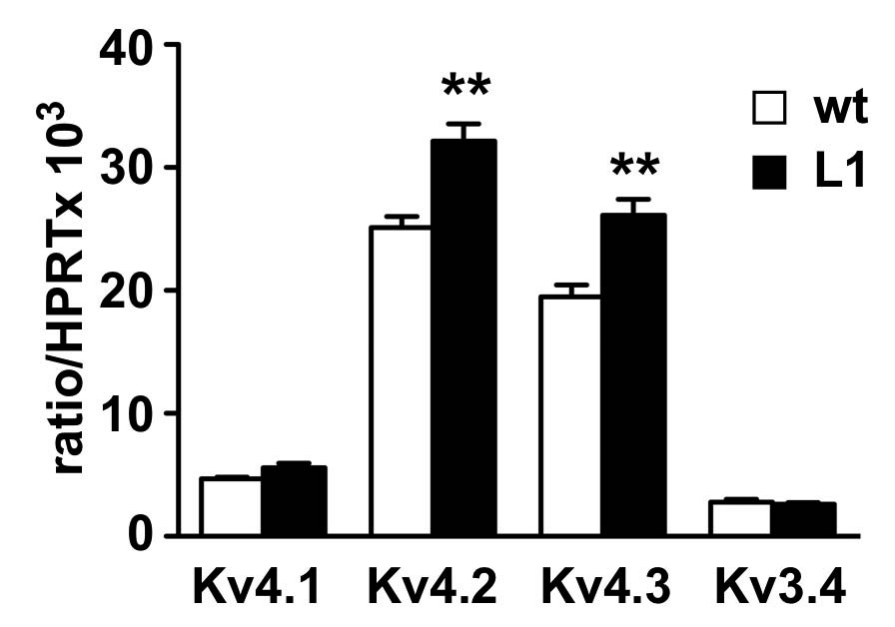
**Expression of Kv4 channels is increased in L1 mice**. Quantitative real-time PCR analysis of the different potassium channels that participate in the generation of A-type currents in the spinal cord. The mRNA levels in wild type (wt) and transgenic L1 mice are expressed as ratio to HPRT mRNA levels. Data are mean ± SEM (n = 8). ** *P *< 0.01.

Quantitative analysis of the mRNA levels of different Kv subunits contributing to A-type currents in spinal cord from P7 mice showed small but significant increases of Kv4.2 and Kv4.3 in daDREAM mice, while Kv4.1 and Kv3.4 levels were unaffected (Figure 5). Reduced expression of Kv4 subunits has been associated with basal hyperalgesia [15]. In L1 mice, the hyperalgesia was observed in the presence of a moderate up-regulation of Kv4 subunits, which however did not lead to significant changes in A-type currents recorded in dorsal horn neurons.

### Lack of spinal sensitization in L1 mice following inflammation and low frequency stimulation of C-fibres

**Figure 6:**
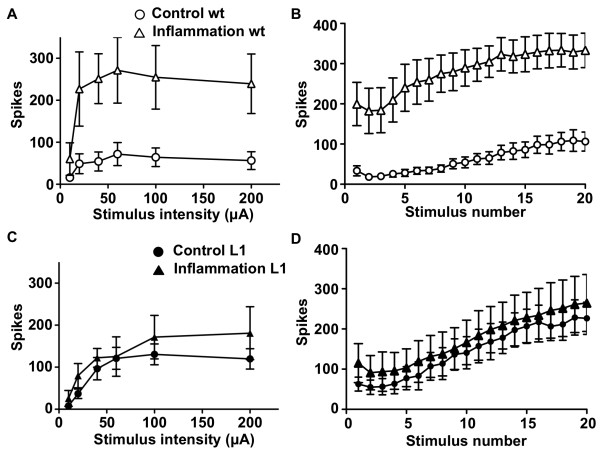
**L1 mice lack sensitization**. (A and C) The number of spikes in ventral root responses to single dorsal root stimuli of increasing intensities and (B and D) to trains of C-fiber intensity stimuli is shown. Responses in spinal cords from non-treated (Control) and carrageenan-treated (Inflammation) wild type mice (wt) and L1 mice are shown. Inflammation produced significantly increased responses in wild type (n = 6, overall ANOVA *P *< 0.01), but not in L1 (n = 5) spinal cords.

Since the hyperalgesic response to peripheral inflammation is reduced in L1 mice, we analyzed changes in spinal cord function after inflammation. Spinal cords from carrageenan-treated wild type P9 mice showed large and significant increases in DR-VRRs to single (Figure 6A) and repetitive stimuli (Figure 6B) compared to nontreated wild type mice. In contrast, the same treatment failed to produce differences in spinal reflexes in L1 mice (Figure 6C, D).

**Figure 7:**
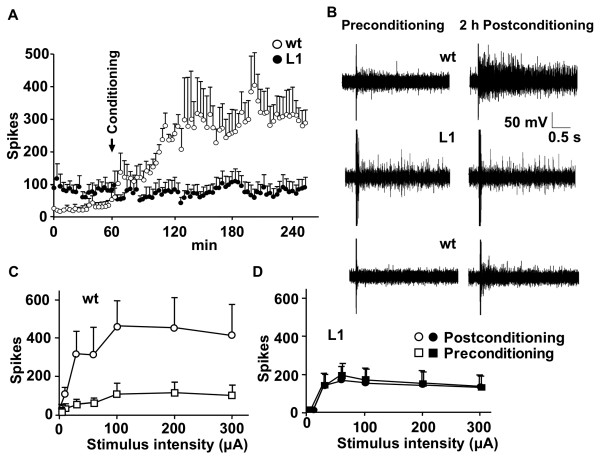
**L1 mice lack spinal cord potentiation**. (A) Number of spikes elicited by A-fiber intensity stimuli before and after administration of a C-fiber specific conditioning stimulus consisting of 240 shocks (200 μA, 200 μs at 2 Hz). Wild type (wt) (n = 5) mice developed a large and significant increase (One-way ANOVA, Bonferroni post-test, *P *< 0.01), whereas L1 (n = 6) mice did not show any change in the number of elicited spikes. (B) Representative original recordings obtained from wild type (wt) and L1 mice before and 2 h after C-fiber conditioning. Bottom panel, shows a representative original recording of the absence of potentiation in wild type mice 2 h after A-fiber conditioning. (C and D) Number of spikes in ventral roots by graded dorsal root stimulation immediately before (Preconditioning) and 2 h after conditioning (Postconditioning), obtained from wild type (wt) (n = 5) and L1 (n = 6) mice.

In order to model *in vitro *the central sensitization induced by inflammation in wild type mice, we used a protocol of low frequency stimulation of the dorsal roots similar to that reported to produce LTP in superficial dorsal horn neurons [28]. In isolated spinal cords from wild type mice, application of a conditioning protocol consisting of a low frequency and prolonged stimulation of C-fibres resulted in long-term enhancement of DR-VRRs (Figure 7A, B), whereas application of an identical conditioning protocol at A-fiber intensity did not produce changes in DR-VRRs (Figure 7B, bottom panel). The enhancement developed slowly within the first hour post-conditioning, reached a plateau between 2 and 3 h and was still observed 4 h after application of the conditioning stimulus, the longest recorded period. In L1 mice, application of the conditioning stimuli at C-fibre intensity did not produce the enhancement of DR-VRRs (Figure 7A, B). After inflammation, persistent stimulation of C-fibres increased ventral responses to dorsal root stimulation of different intensities in spinal cords from wild type mice (Figure 7C) but not in transgenic spinal cords (Figure 7D), indicating that L1 mice are unable to generate central sensitization.

### BDNF mediates long-term enhancement of DR-VRRs

BDNF is believed to play an important role in spinal plasticity and sensitization following carrageenan-inflammation [29]. Therefore, we investigated whether reduced spinal levels of BDNF could be responsible for the lack of central sensitization observed in transgenic mice.

**Figure 8:**
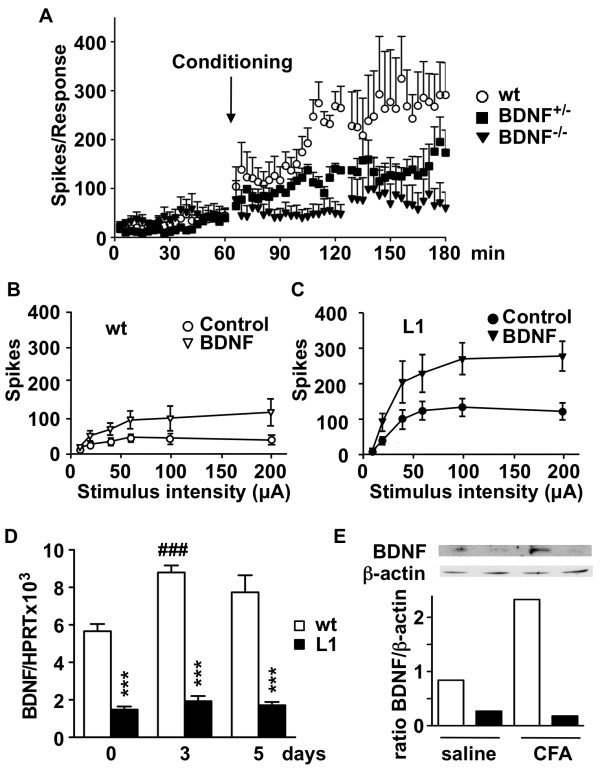
**The levels of BDNF in L1 mice are not sufficient for spinal sensitization**. (A) Time course for ventral root responses to a series of A-fiber intensity stimuli, before and after C-fiber specific conditioning (200 μA, 200 μs at 2 Hz). Data were obtained from spinal cords of wild type (wt) (n = 6), BDNF^+/- ^(n = 3) and BDNF^-/- ^(n = 5) mice. In the BDNF^+/- ^group, DR-VRRs were larger in the 2 h postconditioning compared to the preconditioning period (One-way ANOVA, Bonferroni post-test, *P *< 0.05). In the BDNF^-/- ^group the conditioning stimulus failed to modify DR-VRRs. (B and C) Ventral root responses by graded stimuli applied to the dorsal root before and after prolonged superfusion of exogenous BDNF, obtained from wild type (wt) (n = 5) and L1 (n = 6) mice. (D) BDNF mRNA levels in DRG were measured by quantitative PCR before, 3 and 5 days after intraplantar injection of complete Freund's adjuvant in wild type (wt) (n = 6) and L1 (n = 6) adult mice. The mRNA levels are expressed as ratio to HPRT mRNA levels. * Refers to comparison with wild type and # refers to comparison with day 0. Statistically significant differences between groups were measured by One-way ANOVA with Bonferroni post-test. *** and ###, *P *< 0.001. (E) BDNF protein levels in lumbar spinal cord 36 h after intraplantar injection of saline or complete Freund's adjuvant in wild type (wt) (n = 3+3) and L1 (n = 3+3) adult mice. Pools of the three mice in each experimental group were analyzed. The immunoreactive bands and densitometry quantification are shown. BDNF values were corrected by β-actin levels.

First we analyzed whether the presence of BDNF is necessary for segmental spinal circuits to generate a long-term enhancement of DR-VRRs in response to the prolonged low frequency C-fibre stimulation protocol. For this, we used BDNF^-/- ^and BDNF^-/+ ^P9 mice. Isolated spinal cords from BDNF^-/- ^mice did not respond to the C-fibre conditioning protocol, while those from BDNF^-/+ ^mice showed a deficient enhancement of DR-VRRs (Figure 8A).

We then analyzed changes in DR-VRRs before and after exogenous applications of BDNF to spinal cords of wild type and L1 mice. Consistent with our previous result that TrkB receptor mRNA levels are not modified in L1 mice, the response to exogenous addition of BDNF was similar in spinal cords from wild type and L1 mice (Figure 8B, C). In both cases, BDNF superfusion produced a significant 2-fold increase in spikes associated to the DR-VRRs elicited by a wide range of stimulus intensities involving activation of A- and C-fibres.

These results indicate that BDNF is essential for the development of spinal sensitization and suggest that sensitization would occur in L1 mice if they could mobilize enough BDNF in response to persistent activation of nociceptive afferents i.e. chronic inflammation or in response to injury. To test this hypothesis we analyzed the expression of BDNF mRNA in DRG and BDNF protein in spinal cord following the application of CFA. Importantly, the inflammatory response in wild type mice developed with an increase in BDNF mRNA levels that was maximal at 3 days and remained elevated 5 days after CFA injection (Figure 8D). In DRG from L1 mice, however, levels of BDNF mRNA were not increased at any time after CFA injection (Figure 8D). Importantly, western blot analysis of BDNF protein levels in spinal cord confirmed the mRNA data and showed a 70% decrease in BDNF protein levels in transgenic spinal cord and the absence of induction upon inflammation (Figure 8E). Conversely, a substantial increase in BDNF protein was readily observed in wild type mice upon inflammation (Figure 8E).

## Discussion

The dominant active mutant, daDREAM, blocks the regulated activity of endogenous DREAM/KChIP proteins when transgenic to endogenous mRNA ratio is as low as 1 to 6 [7]. In this study, we show that in L1 transgenic mice the ratio daDREAM/DREAM mRNA is 1.6 to 1 and 1 to 3 in spinal cord and DRG, respectively, indicating that the expression of the dominant active mutant protein is sufficient to block Ca^2+^- and cAMP-mediated derepression by endogenous DREAM/KChIP proteins. As a result, expression of prodynorphin and BDNF, two potential targets for transrepression by DREAM that are related to pain, are reduced in DRG and spinal cord from L1 transgenic mice. Since the mutation at the LCD domain in daDREAM prevents its interaction with CREB [6], downregulation of prodynorphin and BDNF in L1 mice is not related to interference with CREB-dependent transcription and is due to direct repression by daDREAM. Importantly, changes in nociception are related to the expression of the transgene in spinal cord and DRG neurons, since sensory thresholds were normal in L26 mice in which daDREAM expression is confined exclusively to telencephalic areas. In the same way, another line of daDREAM mice, L33, without transgene expression in spinal cord and DRG neurons, did not show any change in pain sensitivity to thermal stimulation [30].

Previous work with DREAM^-/- ^mice [12] demonstrated the involvement of this transcriptional repressor in pain modulation. Mice lacking DREAM showed a basal state of analgesia and reduced response to inflammatory and neuropathic treatments, that was interpreted as caused by elevated spinal cord levels of dynorphin A peptide observed in these mice [12]. Consistent with this, the reduced expression of prodynorphin in L1 mice could account for the basal state of hyperalgesia found in adult mice. The basal hyperalgesia was paralleled by increased hyperreflexia in the isolated spinal cord without signs of change in the function of sensory afferents, suggesting that the exaggerated sensitivity is related to changes in spinal processing of afferent signals.

Activation of NMDA receptors plays a central role in the transmission of primary sensory information in the spinal cord [reviewed in [31]]. Recently, it has been shown that DREAM reduces the amplitude of NMDA currents in hippocampal neurons through a Ca^2+^-sensitive interaction between DREAM and PSD95 [30] or between DREAM and the NR1 subunit of the receptor [32]. On the other hand, it was previously shown that release of dynorphin peptides from presynaptic terminals inhibits glutamatergic trasmission through NMDA receptors [33]. The increased hyperreflexia in isolated spinal cord and the increased basal sensitivity to pain stimulation suggest that a potential reduction in NMDA currents in spinal cord neurons in daDREAM transgenic mice is compensated by the reduced inhibition due to the low expression of prodynorphin.

In addition to the hypothesis of prodynorphin involvement, there is a convergence of data suggesting that transient A-type potassium currents, mediated by Kv4 channels, are responsible for hypersensitivity to acute pain stimuli. Genetic elimination of Kv4.2 reduces A-type currents and increases excitability of dorsal horn neurons, resulting in enhanced sensitivity to noxious stimuli [15], that resembles the scenario in L1 mice. Moreover, genetic ablation of Kv channels results in decreased expression of DREAM/KChIP proteins [34], suggesting a genetic auto-regulatory loop between these two gene families. Our results, however, are not consistent with the hypothesis that reduced expression of Kv channels in the spinal cord or malfunction of the associated currents, are responsible for the altered noxious sensitivity in L1 transgenic mice. On the contrary, we found small but significant increase in Kv4.2 and Kv4.3 mRNA levels in the spinal cord and, more important, we found that I_A _currents in dorsal horn neurons from L1 mice were indistinguishable from those of wild type mice in terms of various properties, including current density and kinetics.

Reduced prodynorphin levels may explain basal hypersensitivity of L1 mice, however, does not account for the reduced behavioral response to inflammation following CFA injection. Importantly, isolated transgenic spinal cords were not capable to show signs of central sensitization, which may be causal for the anomalous response to inflammatory stimuli. Whereas spinal cords from carrageenan-treated wild type mice showed a marked hyperreflexia, L1 mice with or without treatment, showed essentially similar spinal reflexes. Furthermore, persistent low frequency stimulation of primary C-afferents caused a long-term enhancement of DR-VRRs in wild type but not in L1 mice. It is worth noting, that similar protocols of stimulation [35] have been shown to produce LTP in superficial laminae neurons in a process that resembles central sensitization induced by inflammation.

In order to understand how L1 mice fail to produce a normal process of central sensitization, we considered the reduced expression levels of BDNF in L1 mice. Reduced BDNF levels *in vivo *confirm previous *in vitro *studies showing the regulatory effect of DREAM on BDNF promoter activity [9]. Several lines of evidence relate BDNF with central sensitization; i) intrathecal administration of exogenous BDNF produces hyperalgesia in wild type mice, whereas administration of antiserum directed against either BDNF or TrkB receptors prevent inflammation-induced hyperalgesia [29,36], ii) conditional BDNF knockout mice do not develop hyperalgesia after inflammatory stimuli [37] and iii) at the functional level, BDNF has been shown to be required for simple forms of spinal reflex plasticity like wind-up [38] and to enhance the spinal response to sensory inputs [16,39] through post-synaptic NMDA receptors [16] or by reversing chloride gradients in central afferent terminals [17]. Here we show that BDNF is required also for longer lasting forms of plasticity in spinal reflexes. BDNF^-/- ^mice did not develop long-term enhancement of ventral root reflexes following low frequency stimulation of C-fibers. Interestingly, BDNF^-/+ ^mice were able to develop a small amplitude enhancement of reflexes, suggesting that the quantitative expression of BDNF in the spinal cord is correlated with the ability to develop long-term forms of spinal plasticity.

DREAM transgenic mice express normal levels of TrkB receptors in the spinal cord and therefore we were able to test the effects of exogenous applications of BDNF to the isolated spinal cord. BDNF produced a similar enhancement of spinal responses to afferent inputs in spinal cords from wild type and L1 mice, suggesting that the spinal cord from L1 mice does not receive enough BDNF from primary afferents after low frequency stimulation of C-fibers or after inflammation. The absence of induction in BDNF expression in dorsal root ganglia from L1 mice following inflammation supports this idea.

## Conclusions

Transgenic L1 mice show a state of basal hyperalgesia most likely sustained by low spinal cord levels of dynorphin and which is not related to anomalies in Kv expression or function. More importantly, our work suggests that the reduced hyperalgesic response in L1 mice following peripheral inflammation and the reduced central sensitization is mostly due to deficient BDNF input in the spinal cord from primary afferents.

## Methods

### Animals and behavioral analysis

Experiments were performed with C57B/6×CBA mice. BDNF^-/- ^mice were kindly provided by Dr. J. Alberch (University of Barcelona). The generation of DREAM transgenic mice has been described [7]. Experiments were performed in adult male mice, homozygous for the transgene (line L1) and in wild type mice. Mice were housed five per cage in a temperature (21 ± 1°C) and humidity (65 ± 10%) controlled room with a 12-/12- h light/dark cycle (lights on from 8 am to 8 pm hours) with food and water *ad libitum*. Experiments took place during the light phase. Behavioral tests and animal care were conducted in accordance with the standard ethical guidelines (European Communities Directive 86/609 EEC; National Institutes of Health 1995) and approved by the local ethical committee (CNB-UAH). All experiments were carried out in blind conditions. Behavioral testing by plantar test, tail flick, writhing test and von Frey hairs were performed as described [24,40-42]. Inflammation was induced by unilateral intraplantar injection of carrageenan (30 mg/ml in saline; 20 μl injection volume) or complete Freund adjuvant (50 μl) in P6-10 or adult mice.

### RNA extraction and quantitative PCR

RNA from wild type and L1 spinal cord and dorsal root ganglia was prepared using TRIzol (Invitrogen) and the RNAeasy Mini Kit (Qiagen). RNA was quantified and the quality was assessed with a 2100 Bioanalyzer (Agilent). Quantitative real-time PCR for endogenous DREAM and mutant daDREAM was performed using a pair of primers able to amplify both: forward 5'-CACCTATGCACACTTCCTCTTC A-3' and reverse 5'-ACCACAAAGTCCTCAAAGTGGAT-3' and two TaqMan MGB probes; FAM-5'-TGCCTTCGATGCTGAT-3'-MGB and VIC-5'-CGCCTTTGCTGCGGC-3'-MGB, specific for DREAM and daDREAM, respectively. The results were normalized by quantification of HPRT mRNA using the specific primers; forward 5'-TTGGATACAGGCC AGACTTTGTT-3' and reverse 5'-CTGAAGTACTCATTATAGTCAAGGGCATA-3', and the probe FAM-5'-TTGAAATTCCAGACAAGTTT-3'-MGB. Quantitative PCR for prodynorphin, BDNF and TrkB were performed using kits from Applied Biosystems.

### Western blot analysis

Lumbar segments (L4 and L5) were dissected, frozen at -70°C and homogenized in extraction buffer [100 mM PIPES, pH 7.0; 0.5 M NaCl; 0.2% Triton-X 100; 2% BSA; 2 mM EDTA; protease inhibitors Complete Mini (Roche)]. After centrifugation at 16,000× g for 30 min, spinal cord extracts were resolved in SDS-PAGE and transferred to PVDF membranes (Millipore). Antibodies against β-actin (clone AC-15, Sigma) and BDNF (affinity purified sheep anti-BDNF, OSB00026A, Osense) were used and blots were developed using ECL (Supersignal West Dure, Pierce). Band intensity were quantified using the software QuantityOne (Biorad) and related to β-actin to correct for protein loading.

### In vitro spinal cord preparation and dorsal root stimulation

Wild type and transgenic (L1) 6-11 days-old mice of either sex weighing between 4.3 and 9.7 g were anaesthetized with urethane (2 g/Kg i.p.) and spinal cords were extracted following a rostrocaudal laminectomy [43]. Entire or hemisected spinal cords (for ventral root or single cell recordings) were pinned down to a Sylgard-based recording chamber and maintained with oxygenated artificial cerebrospinal fluid (ACSF) at room temperature (22 ± 1°C). The composition of the ACSF was: (in mM) NaCl 127, KCl 1.9, KH_2_PO_4 _1.5, MgSO_4 _1.3, CaCl_2 _2, NaHCO_3 _22, glucose 10, (pH 7.4). One lumbar dorsal root (L4-L5) was placed in a tight fitting glass suction electrode and electrically stimulated to activate afferent fibers. Single stimuli of 200 ms duration were applied at a range of intensities to activate A-fibers or all fibers in the dorsal root [26]. Wind-up stimuli consisted of 20 C-fiber intensity shocks (200 μs, 200 μA) applied at 1 Hz. Conditioning stimuli to produce a long-lasting enhancement of dorsal root-ventral root reflexes (DR-VRRs) consisted of 240 C-fiber intensity shocks (200 μs, 200 μA) delivered at 2 Hz.

### Ventral root recordings

L4-L5 ventral roots were placed in a suction electrode to record averaged activity from motoneurons. Recordings were made with a Cyberamp (Axon Instruments, CA, USA) in AC mode as previously described [26]. AC recordings showed individual fast events, or spikes or groups of spikes, reflecting the simultaneous firing of action potentials in a group of motoneurons. The number of such events was quantified using amplitude criteria as previously described [44]. Responses to single stimuli were quantified as the number of spikes counted in a time window between 20 ms and 4 s from the stimulus artifact. Responses to trains of stimuli were quantified as the number of spikes within 20 ms and 0.95 s after each stimulus artifact of the train.

### Current and voltage clamp recordings

Electrodes were pulled from borosilicate glass tubing with internal filament using a horizontal puller (Sutter instruments, Novato, CA, USA) giving resistances within the range of 6-9 MΩ. Internal solution consisted of: (in mM) KCl 30, EGTA 3, HEPES 40, MgCl_2 _2, potassium acetate 95, CaCl_2 _0.5, Na_2_-ATP 3, Na-GTP 0.3 (pH 7.4). Signals were amplified with a MultiClamp 700A amplifier (Axon Instruments, CA, USA) and analyzed offline with Spike 2 and Signal software (CED, Cambridge Electronic Designs, Cambridge, UK). Electrode tracking as well as current and voltage recordings were performed using protocols and procedures explained in full elsewhere [45]. Transient potassium currents sensitive to 4-aminopyridine and insensitive to tetraethyl ammonium were isolated in the presence of 0.5 μM tetrodotoxin to block sodium currents and 100 μM cadmium chloride to block calcium and calcium-activated potassium currents as previously described [46,47].

### Drugs and chemicals

Carrageenan lambda, Complete Freund Adjuvant (CFA), cadmium chloride (CdCl_2_), tetraethyl ammonium (TEA), 4-aminopyridine (4-AP) and the components for the ACSF and the intracellular solution were from Sigma-Aldrich (Spain). Tetrodotoxin (TTX) was from Tocris Bioscience (Bristol, UK) and BDNF from Peprotech (CA, USA).

### Data analysis

Statistical analysis and curve fittings were performed using Prism 4.0 (GraphPad Software, USA). Differences between pairs of mean values were analyzed using the Mann-Whitney test or One-way ANOVA. Responses to dorsal root stimulation and intracellular pulses were quantified in terms of number of spikes against intensity of dorsal root stimulus or intracellular pulse respectively, and the resulting curves were analyzed using Two-way ANOVA. Data of normalized conductance, activation and inactivation curves were treated as previously reported [45]. Curves were compared by Two-way ANOVA followed by Bonferroni post-tests. Data is expressed as mean ± SEM, unless otherwise stated.

## Competing interests

The authors declare that they have no competing interests.

## Authors' contributions

IRA carried out electrophysiological recordings. TB carried out behavior analysis, RNA extraction and quantitative PCR. CR carried out behavior analysis and electrophysiological recordings. BT participated in RNA extraction and quantitative PCR. JB and AK participated in behavior analysis. CA participated in analysis and interpretation of data, drafting, critical revising and final approval of the manuscript. BM carried out analysis and interpretation of data, drafting, critical revising and final approval of the manuscript. JALG and JRN carried out conception and design, analysis and interpretation of data, drafting, critical revising and final approval of the manuscript. All authors read and approved the final manuscript.
